# {Fe_6_O_2_}-Based Assembly of a Tetradecanuclear Iron Nanocluster

**DOI:** 10.3390/ma4010300

**Published:** 2011-01-19

**Authors:** Svetlana G. Baca, Manfred Speldrich, Arkady Ellern, Paul Kögerler

**Affiliations:** 1 Institute of Inorganic Chemistry, RWTH Aachen University, 52074 Aachen, Germany; E-Mails: sbaca_md@yahoo.com (S.G.B.); manfred.speldrich@ac.rwth-aachen.de (M.S.); 2 Institute of Chemistry, Academy of Sciences of Moldova, MD2028 Chisinau, Moldova; 3 Ames Laboratory, Iowa State University, Ames, IA 50011, USA; E-Mail: ellern@iastate.edu; 4 Institute of Solid State Research, Research Centre Jülich, 52425 Jülich, Germany

**Keywords:** Iron(III) carboxylates, Solvothermal synthesis, X-ray structure, Magnetic properties

## Abstract

The tetradecanuclear Fe^III^ pivalate nanocluster [Fe_14_O_10_(OH)_4_(Piv)_18_], comprising a new type of metal oxide framework, has been solvothermally synthesized from a hexanuclear iron pivalate precursor in dichlormethane/acetonitrile solution. Magnetic measurements indicate the presence of very strong antiferromagnetic interactions in the cluster core.

## 1. Introduction

Polynuclear iron coordination clusters have received considerable attention since the discovery that they can serve as models for protein active sites [1] and exhibit interesting magnetic properties including slow magnetization relaxation and quantum tunneling phenomena [2,3,4,5,6,7,8,9,10,11,12,13,14]. Tremendous efforts have been devoted to the development of new synthetic routes to increase the nuclearity of such clusters. One synthetic strategy is based on serendipitous self-assembly of ferric salts and simple ligands, which allows for a balance between the preferred coordination geometry of the metal ions and the coordination mode of the ligand [15]. The vast majority of single molecular magnets have been made in such a serendipitous manner. A second approach involves reactions of small metal cages (typically oxo-centered homo- and heterotrinuclear complexes) in solution (including the use of non-coordinating, high-boiling solvents such as decane or tetradecane) or at high temperature in the solid state. A survey of compounds derived in this manner indicates that such compounds can eliminate weakly bound solvent molecules or ligands, giving higher nuclearity metal clusters by oligomerization of the initial metal cores [16,17,18,19]. A third, very useful synthetic path includes the use of hydrolysis or alcoholysis reactions in the presence of carboxylate groups [20,21]. In the forth synthetic strand, hydro- and solvothermal heating under higher pressure as well as a microwave irradiation has also proven useful to induce aggregation of smaller units into larger clusters, with several reported precedents [22,23]. Other approaches to high-nuclearity or high-symmetry Fe*_n_* polytopes utilize diamagnetic polyoxometalate scaffolding structures [24,25].

Herein we describe the preparation of the neutral tetradecanuclear Fe(III) oxo/hydroxo-based cluster [Fe_14_O_10_(OH)_4_(Piv)_18_] (**1**) (where HPiv = pivalic acid) by a stepwise exploration of different synthetic approaches: starting from ferric nitrate salt in the excess of pivalic acid to produce a μ_3_-oxo trinuclear iron(III) pivalate species, [Fe_3_O(Piv)_3_(H_2_O)_3_] Piv · 2HPiv [17], which then aggregates to a hexanuclear [Fe_6_O_2_(OH)_2_(Piv)_12_] pivalate cluster [17,26] under heating in tetradecane, and then converting this hexanuclear Fe^III^ cage into higher tetradecanuclear ferric cluster under solvothermal conditions. To our knowledge, only few tetradecanuclear Fe^III^ clusters are known. These include triazole-bridged [Fe_14_O_6_(L)_6_(OMe)_18_Cl_6_] clusters (where L = 1,2,3-triazole, benzotriazole, 5-methylbenzotriazole, 5,6-dimethylbenzotriazole, 5-chlorobenzotriazole) [22,27] with a {Fe_14_O_6_} core, an alkoxide-based [Bu_4_N]_2_[Fe_14_O_8_(OCH_2_Me)_20_Cl_8_] [28] cluster and a oxo/peroxo pivalate-type [Fe_14_O_4_(O_2_)_2_(Piv)_12_(L)_8_(H_2_O)_12_](NO_3_)_2_ cage (where L = phosphonate ligand) [29] possessing a {Fe_14_O_8_} core, and finally a nitride (Et_4_N)_4_[Fe_14_N_8_(L)Cl_12_] (where L = (Me_3_Sn)_3_N) cluster [30] with a {Fe_14_N_8_} core, yet no tetradecanuclear cluster with a {Fe_14_O_14_} core has been reported.

## 2. Results and Discussion

### 2.1. Synthesis and Preliminary Characterization

**Scheme 1:**
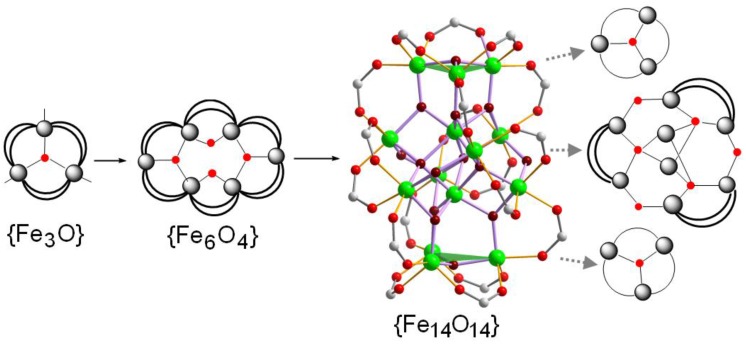
Synthesis of [Fe_14_O_10_(OH)_4_(Piv)_18_] (**1**). The simplified ball-and-stick representation of **1** is reduced to the {Fe_14_O_14_} core with 18 adjoined carboxylate groups. *tert*-Butyl groups and hydrogen positions are omitted for clarity. Fe: green spheres, O(carboxylate): red, C: light grey, O (Fe_14_O_14_ fragment): dark red. Fe−O bonds of the central {Fe_14_O_14_} core shown in light purple, Fe−O(carboxylate) in orange. Two green triangles highlight the position of the top and bottom Fe_3_(µ_3_-O) groups.

The tetradecanuclear cluster [Fe_14_O_10_(OH)_4_(Piv)_18_] (**1**) has been prepared by several steps, as shown in Scheme 1, starting from the well-known μ-oxo trinuclear pivalate precursor [Fe_3_O(Piv)_3_(H_2_O)_3_]Piv·2HPiv synthesized by Gerbeleu *et al.* in 1987 [17]. Heating of the μ-oxo trinuclear pivalate in tetradecane led to a hexanuclear pivalate cluster [Fe_6_O_2_(OH)_2_(Piv)_12_], initially prepared by the same group [17,24]. The treatment of the [Fe_6_O_2_(OH)_2_(Piv)_12_] cluster in a dichlormethane/acetonitrile solution at 120 °C for 4 hours in a PTFE-lined stainless steel autoclave followed by slow cooling to room temperature over 48 hours results in **1** in *ca.* 50% yield.

The infrared spectrum of **1** displays strong and broad bands in the 1590–1547 cm^–1^ region and at 1429 cm^–1^, arising from asymmetric and symmetric stretching vibrations of the coordinated carboxylate groups of the pivalate ligands, respectively. The C–H asymmetric and symmetric stretch vibrations for *tert*-butyl groups of pivalates are observed in the 2962–2872 cm^–1^ region, along with a strong single band at 1485 cm^–1^ and a doublet at 1379–1362 cm^–1^, which correspond to asymmetric and symmetric bending vibrations for methyl groups, respectively. The weak bands at 3633 and 3610 cm^–1^ are ν(OH) vibrations.

Its neutral state and the highly lipophilic cluster surface of **1**, nearly completely defined by the *tert*-butyl groups, renders the cluster soluble in *e.g.*, dichloromethane or tetrahydrofurane but insoluble in water.

### 2.2. X-ray Structure

**Figure 1:**
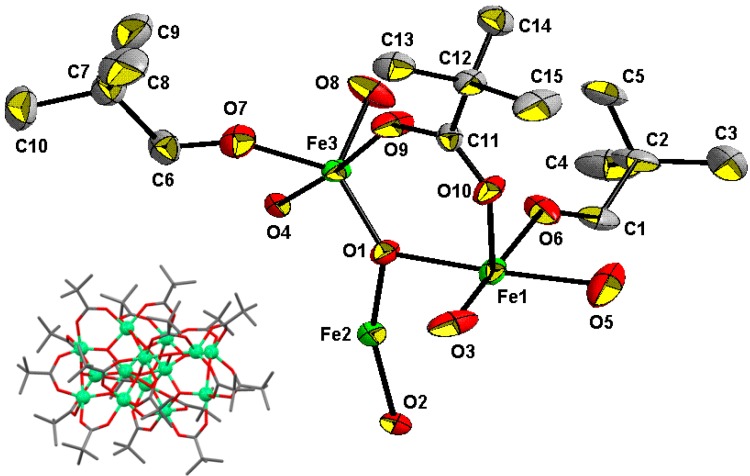
Asymmetric unit in [Fe_14_O_10_(OH)_4_(Piv)_18_] (**1**) with atom labels. The anisotropic displacement ellipsoids are set at 50% probability. Inset: molecular structure of **1**. Hydrogen atoms are omitted for clarity. Color scheme: Fe, green; O, red; C, grey.

**Figure 2:**
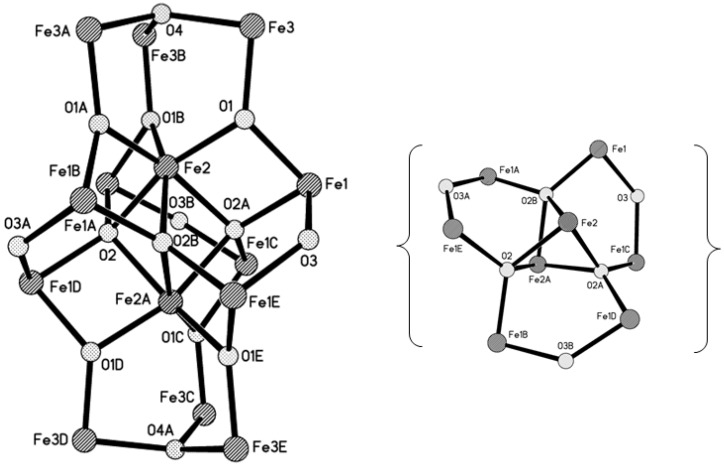
The {Fe_14_O_14_} core in [Fe_14_O_10_(OH)_4_(Piv)_18_] (**1**). All pivalate ligands are omitted for clarity. The inset shows a top-down view of the central {Fe_8_O_6_} fragment.

Cluster **1** crystallizes in the hexagonal space group *R-3c* with *a* = 15.6604(17), *b* = 15.6604(17), *c* = 96.907(13) Å. An ORTEP drawing of the asymmetric unit in **1** with the labeling scheme and the complete molecular structure of **1** are shown in Figure 1. Selected bond distances and angles of **1** are listed in Table 1. The structure of **1** consists of 14 Fe^III^ atoms (there are only three crystallographically independent Fe atoms, Fe1, Fe2 and Fe3), which are connected by three μ_4_-oxo atoms, eight μ_3_-oxo atoms and three μ_2_-hydroxo atoms forming a [Fe_14_(μ_4_-O)_3_(μ_3_-O)_8_(μ_2_-OH)_3_] core, as shown in Figure 2, and 18 bridging pivalate ligands at the periphery of the molecule.

**Figure 3:**
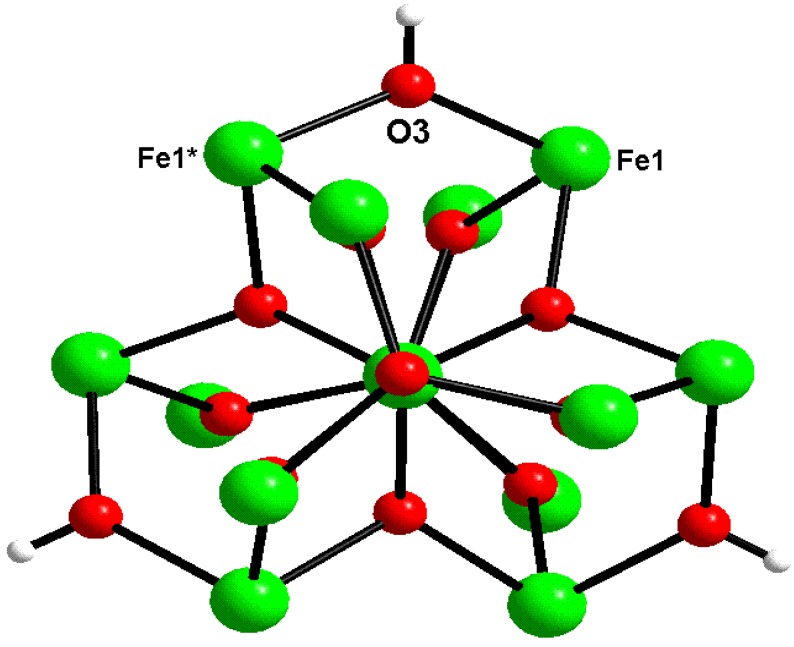
Top view of metallic core in [Fe_14_O_10_(OH)_4_(Piv)_18_] (**1**) highlighting the μ_2_-hydroxo groups. All pivalate ligands are omitted for clarity.

The metallic core (Figure 2) can be divided into three layers of two types. The upper and bottom layers are Fe_3_ trinuclear units (Fe3, Fe3A and Fe3B and Fe3C, Fe3D and Fe3E, respectively) with a central μ_3_-O atom (O4 and O4A, respectively) in the center and three bridging pivalates. The Fe···Fe distances in this trinuclear unit are all equal to 3.298(1) Å. The middle layer consists of eight Fe atoms (Fe1 and Fe2 atoms and their equivalents) held together by three μ_4_-O atoms (O2, O2A, O2B) to form three [Fe_4_(μ_4_-O)] tetrahedra sharing the common Fe2−Fe2A edge with the shortest Fe···Fe distance of 2.799(2) Å (Table 1). A pair of pivalate ligands additionally connects the outer Fe atoms (Fe1 and its equivalents) in each tetrahedron. This Fe_8_ arrangement resembles a propeller with three tilted blades. The three μ_2_-OH atoms (O3, O3A, O3B) (Figure 3) link the blades of the propeller. The outer trinuclear groups are bridged to the octanuclear iron layer via μ_3_-O positions (O1 and its equivalents) and three pivalate ligands, resulting in three additional Fe_3_ trinuclear units with central μ_3_-O atoms (O1 and its symmetry equivalents) and one bridging pivalate. As for the Fe···Fe distances within the triangle, the Fe2···Fe1 distance of 2.962(7) Å is significantly shorter than the Fe1···Fe3 (3.464(1) Å) and Fe2···Fe3 (3.376(1) Å) distances.

**Table 1:** Selected bond distances (Å) and angles (°) in **1**.

Fe1−O2A	1.965(2)	Fe2−O1B	1.951(3)
Fe1−O3	1.966(2)	Fe2−O2B	2.090(3)
Fe1−O1	1.970(3)	Fe2−O2A	2.090(3)
Fe1−O10	2.021(3)	Fe3−O1	1.856(3)
Fe1−O5	2.033(4)	Fe3−O4	1.934(1)
Fe1−O6	2.033(4)	Fe3−O7	1.958(3)
Fe2−O1	1.951(3)	Fe3−O8	1.975(3)
Fe2−O1A	1.951(3)	Fe3−O9	2.004(3)
Fe2−O2	2.091(3)		
Metal···Metal separations			
Fe1···Fe3	3.4638 (11)	Fe2···Fe2C	2.799(2)
Fe2···Fe1	2.962(7)	Fe2···Fe3	3.3756 (11)
Fe3···Fe3A	3.2983 (11)		

O2A−Fe1 −O3	93.01(13)	O1−Fe2−O2B	98.78(10)
O2A−Fe1 −O1	85.08(11)	O1A−Fe2−O2B	82.26(10)
O3 −Fe1 −O1	94.58(11)	O1B−Fe2−O2B	162.24(10)
O2A−Fe1 −O10	177.44(12)	O1−Fe2−O2A	82.26(10)
O3−Fe1−O10	87.04(15)	O1A−Fe2−O2A	162.24(10)
O1−Fe1−O10	92.37(12)	O1B−Fe2−O2A	98.78(10)
O2A−Fe1−O5	96.96(14)	O2B−Fe2−O2A	80.09(11)
O3−Fe1−O5	88.05(16)	O1−Fe2−O2	162.24(10)
O1−Fe1−O5	176.60(17)	O1−Fe3−O4	93.91(16)
O10−Fe1−O5	85.60(14)	O1−Fe3−O7	122.30(15)
O2A−Fe1−O6	93.15(11)	O4−Fe3−O7	92.93(13)
O3−Fe1−O6	172.60(16)	O1−Fe3−O8	132.27(15)
O1−Fe1−O6	90.03(14)	O4−Fe3−O8	90.90(13)
O10−Fe1−O6	86.98(14)	O7−Fe3−O8	104.78(16)
O5−Fe1−O6	87.14(18)	O1−Fe3−O9	92.77(13)
O1−Fe2−O1A	98.62(11)	O4−Fe3−O9	173.22(17)
O1−Fe2−O1B	98.62(11)	O7−Fe3−O9	84.43(14)
O1A−Fe2−O1B	98.62(11)	O8−Fe3−O9	83.76(14)
*A: –y*+1, *x–y*, *z B: –x+y*+1, *–x*+1, *z C: y*+1/3, *x*–1/3, –*z*+1/6			

In the middle layer the eight Fe atoms are all hexa-coordinated with an O_6_ atom set: two inner Fe^III^ ions (Fe2 and Fe2A) are coordinated by three μ_4_-O with an Fe−μ_4_-O distance of 1.951(3) Å and three μ_3_-O atoms with an Fe−μ_3_-O distance of 2.090(3) Å, whereas the four outer Fe^III^ ions (Fe1 and its equivalents) are coordinated by two μ_4_-O [Fe−μ_4_-O, 1.965(2) and 1.970(3) Å], one μ_2_-OH atom [Fe−μ_2_-O, 1.966(2) Å] and three oxygen atoms from three bridging pivalates [Fe−O_carb_, 2.021(3)–2.033(4) Å]. All Fe atoms in the trinuclear unit (upper and bottom layers) are penta-coordinated with an O_5_ set from two μ_3_-O atoms (Fe−μ_3_-O, 1.856(3) and 1.934(1) Å) and three carboxylate O atoms [the Fe−O_carb_ distances range from 1.958(3) to 2.004(3) Å]. The range of Fe-O bond lengths and nearest-neighbor Fe···Fe distances is fully in line with those of other oxo-bridged {Fe_n_O*_m_*} fragments, *e.g.*, the examples mentioned in the Introduction.

Bond valence sum (BVS) calculations for the Fe atoms indicate an oxidation state +III for all iron centers (see Table 2). The total cationic charge (+42) is only partially compensated by the 18 pivalate groups and the 14 oxygen positions (total anionic charge: –46). Electroneutrality of **1** requires formulation of the cluster with four protons, three of which are indicated to reside on the three O3 atoms, for which a bond valence sum value of 1.12 is found (Table 2). Given that no counterions can be found, we postulate that the fourth proton is disordered over other oxygen positions, resulting in a total of four OH groups in the neutral cluster **1**.

**Table 2:** Bond valence sum calculations for Fe and O atoms in **1**.

Atom	Value	Assigned state
Fe1	3.14	Fe^3+^
Fe2	3.02	Fe^3+^
Fe3	3.06	Fe^3+^
O1	1.93	O^2^^−^
O2	1.96	O^2^^−^
O3	1.12	HO^−^
O4	1.87	O^2^^−^
O5	1.92	O^2^^−^
O6	1.93	O^2^^−^
O7	1.94	O^2^^−^
O8	2.04	O^2^^−^
O9	1.95	O^2^^−^
O10	1.99	O^2^^−^

### 2.3. Magnetic Properties

**Figure 4:**
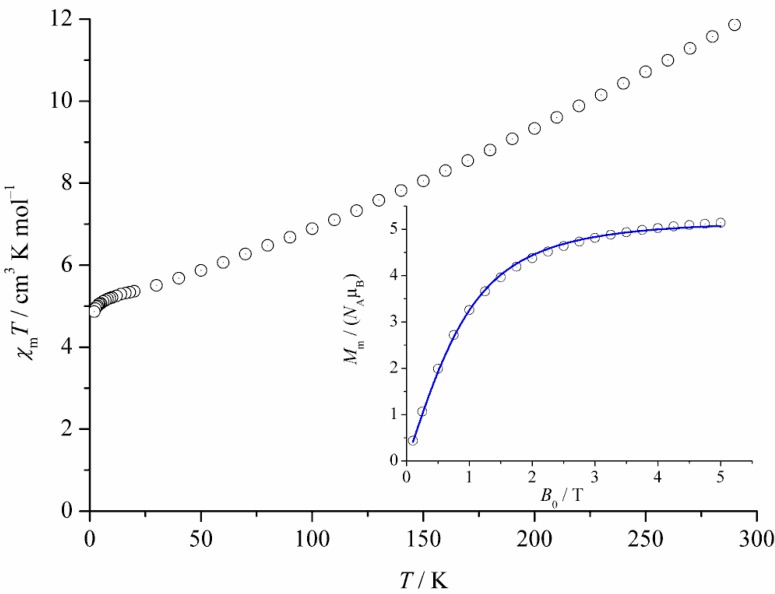
Temperature dependence of χ_m_*T* of [Fe_14_O_10_(OH)_4_(Piv)_18_] (**1**) at 0.1 Tesla (open circles: experimental data). Inset: Field-dependent magnetization at 2.0 K (blue graph: best fit to an *S* = 5/2 Brillouin function).

The presence of all-Fe^III^ ions (high-spin 3d^5^, ^6^A_1g_ ground state for octahedral environments) in **1** simplifies the magnetochemical interpretation as they represent pure spin-5/2 centers. Figure 4 represents the temperature dependence of χ_m_*T* (at 0.1 Tesla) and the isothermal molar magnetization *M*_m_ (at 2 K) as a function of the external field. The *χ*_m_*T vs. T* graph indicates strong dominating antiferromagnetic intramolecular exchange, as expected from the multiple µ-O and µ-OCO exchange pathways in **1**. A plot of the temperature dependence of the reciprocal susceptibility does not approach linear behavior up to 290 K, therefore no Curie-Weiss parameters can be derived. At room temperature, χ_m_*T* approaches a value of 11.86 cm^3^ K mol^–1^, which is much smaller than the spin-only value for 14 isolated Fe^III^ ions considering an average Zeeman factor *g* = 2.0, that is 61.25 cm^3^ K mol^–1^. With decreasing temperature, χ_m_*T* decreases and reaches a shoulder at *ca. T* = 10 K with χ_m_*T* ≈ 5.1 cm^3^ K mol^–1^. This value is higher than the spin-only value obtained for one isolated Fe^III^ ion, *i.e.*, 4.375 cm^3^ K mol^–1^; at lower temperatures, an onset of a steeper decrease in *χ*_m_*T* is apparent. The inset in Figure 4 shows the field dependence of the molecular magnetization cluster up to 5.0 T at 2.0 K. The blue curve is the best fit of the data to the Brillouin function

(1) $$M(H/T)=gS\mu_{B}B(H/T)$$

Least squares fitting indicates that at 2.0 K the magnetic state of **1** can be represented by *S* = 5/2 with the corresponding *g* factor equal to 2.0.

## 3. Experimental Section

### 3.1. Materials and Physical Measurements

All reactions were carried out under aerobic conditions using commercial grade solvents. [Fe_3_O(O_2_CCMe_3_)_6_(H_2_O)_3_]Me_3_CCO_2_·2Me_3_CCO_2_H [17] and [Fe_6_O_2_(OH)_2_(O_2_CCMe_3_)_12_] [17,24] were synthesized as described elsewhere. The infrared spectra were recorded on a Perkin-Elmer Spectrum One spectrometer using KBr pellets in the region 4000–400 cm^–1^. Magnetic susceptibility measurements were performed in a Quantum Design MPMS-5XL SQUID magnetometer as a function of field (0.1 to 5.0 Tesla) and temperature (2.0 to 290 K). Experimental data were corrected for the sample holder (PTFE tubes) and diamagnetic contributions calculated from tabulated values; χ_dia_(**1**) = –1.514 × 10^−3^ cm^3^ mol^–1^.

### 3.2. X-ray Crystallography

Single-crystal X-ray studies were carried out using a Bruker APEX CCD diffractometer employing graphite-monochromated Mo Kα radiation (λ = 0.71073 Å) at 150 K. Details of the crystal, data collection and refinement parameters are in Table 2. The structures were solved by direct methods and refined by full-matrix least squares on weighted |*F*|^2^ values for all reflections using the SHELX program suite. The non-hydrogen atoms were refined with anisotropic displacement parameters. Hydrogen atoms could not be located in difference Fourier maps and were placed in fixed, idealized positions and refined as rigidly bonded to the corresponding atom.

CCDC 803980 contains the supplementary crystallographic data for this paper. These data can be obtained free of charge at www.ccdc.cam.ac.uk/conts/retrieving.html, or from Cambridge Crystallographic Data Center, 12 Union Road, Cambridge, CB2 1EZ, UK (Fax: +44-1233/336-033; E-mail: deposit@ccdc.cam.ac.uk).

### 3.3. Synthesis of [Fe_14_O_10_(OH)_4_(O_2_CCMe_3_)_18_]

[Fe_6_O_2_(OH)_2_(O_2_CCMe_3_)_12_] (0.32 g, 0.2 mmol), CH_2_Cl_2_ (5 mL) and MeCN (5 mL) were placed in a sealed PTFE-lined steel autoclave and heated for 4 hours to 120 °C and then allowed to slowly cool to room temperature over 48 hours. Crystals of **1** were filtered off, washed with MeCN and dried in air (yield: 0.12 g; 49% based on Fe). Elemental analysis calculated (found) for C_90_H_166_Fe_14_O_50_: C, 38.19 (38.40); H, 5.91 (5.97)%. IR (KBr, cm^–^^1^): 3633w, 3610w, 3440br.w, 2962m, 2930m, 2872sh, 1590sh, 1547s, 1485s, 1459s, 1429vs, 1379s, 1362m, 1232s, 1031w, 892w, 787w, 736w, 599s, 551m, 509sh, 485sh, 452m, 438m.

## 4. Conclusions

We have demonstrated how a combination of different synthesis approaches can yield novel polynuclear iron oxide carboxylate cluster structures, as exemplified by a tetradecanuclear Fe^III^ pivalate nanocluster. The cluster comprises a unique {Fe_14_O_14_} core fragment consisting of a condensed {Fe_8_O_6_} building unit in which eight iron atoms are connected by three μ_4_-oxygen atoms and three μ_2_-hydroxy atoms, and two triangular {Fe_3_O} building units attached to the bottom and top of the {Fe_8_O_5_} unit via six μ_3_-oxygen atoms. Eighteen bridging pivalate ligands at the periphery of the molecule additionally stabilize the metal oxide core. Preliminary magnetic susceptibility measurements indicate strong antiferromagnetic exchange caused by both the bridging oxo and carboxylate groups.

Given the well-documented redox activity of archetypal [Fe_3_(µ_3_-O)(µ-RCOO)_6_]*^n^*^+^-type trimers, we expect **1** to also show redox activity, potentially localized at the two outer {Fe_3_(µ_3_-O)(µ-Piv)_3_} trimers but possibly also in its central {Fe_8_O_5_} fragment. Therefore, potential mixed-valent Fe(II/III) derivatives of **1** should in principle exist. In follow-up work, we aim to study the electrochemistry of **1** and probe the synthesis of mixed-valent derivatives by bulk electrolysis as well as by using Fe(II/III) trimer clusters as co-reagents.
